# Creation of a brief group intervention to reduce caregivers burden in an intensive home treatment unit

**DOI:** 10.1192/j.eurpsy.2022.1610

**Published:** 2022-09-01

**Authors:** A. Martín-Blanco, E. Casellas Pujol, L. Gawron Schuster, S. González Simarro, J. Vera Igual, A. Ramírez Guillén, A. Farré Martínez, M. Niubó Cuadras, C. Torres Andreu

**Affiliations:** 1 Hospital de la Santa Creu i Sant Pau. IIB Sant Pau. CIBERSAM., Psychiatry, Barcelona, Spain; 2 Hospital de la Santa Creu i Sant Pau., Psychiatry, Barcelona, Spain; 3 CPB - SSM, Uhpad, Barcelona, Spain

**Keywords:** intensive home treatment, community mental health, caregivers burden

## Abstract

**Introduction:**

Intensive home-treatment (IHT) for people experiencing a mental health crisis has been progressively established in many European countries as an alternative to in-ward treatment. However, the management of acute episodes at home can cause burden in the caregivers of these patients.

**Objectives:**

To create a brief group intervention (BGI) to reduce burden in the caregivers of the patients admitted to an IHT unit.

**Methods:**

A preliminary version of the BGI (BGI 1.0) was designed based on literature’s review. It consisted of 4 sessions of 90 minutes (one per week), on-line (COVID-19), focused on caregivers burden, stress and self-care, communication skills, and self-compassion. All the caregivers of the patients admitted for IHT from 10/01/2020 to 06/01/2021 were offered the BGI 1.0. At the end of the intervention, participants (caregivers and therapists) were asked about their opinion on its contents and usefulness.

**Results:**

A total of 31 caregivers received the BGI 1.0. Most of them felt satisfied with the intervention. Opinions varied as to which contents should be expanded or included. The therapists thought that the number of sessions should be increased to take a closer look at some contents or to include new ones. They also believed that the on-line format hindered the adherence and the interaction between the participants.

**Conclusions:**

The BGI 1.0 seems to be a good starting point to design the final version of the intervention. However, an exhaustive assessment of the construct of burden in a larger sample of caregivers should be performed prior to its design.

**Disclosure:**

No significant relationships.

